# Vaccination With Recombinant Adenoviruses Expressing the Bluetongue Virus Subunits VP7 and VP2 Provides Protection Against Heterologous Virus Challenge

**DOI:** 10.3389/fvets.2021.645561

**Published:** 2021-03-10

**Authors:** José Manuel Rojas, Diego Barba-Moreno, Miguel Avia, Noemí Sevilla, Verónica Martín

**Affiliations:** Centro de Investigación en Sanidad Animal (CISA-INIA), Instituto Nacional de Investigación y Tecnología Agraria y Alimentaria, Madrid, Spain

**Keywords:** vaccine, *Orbivirus*, T cell, cytotoxic T lymphocytes, IFNAR(^−/−^) mice

## Abstract

Bluetongue virus (BTV) is the causative agent of a disease that affects domestic and wild ruminants and leads to critical economic losses. BTV is an arbovirus from the Reoviridae family that is typically transmitted by the bite of infected *Culicoides* midges. BTV possesses multiple serotypes (up to 28 have been described), and immunity to one serotype offers little cross-protection to other serotypes. The design of vaccines that provide protection across multiple serotypes is therefore highly desirable to control this disease. We previously reported that a recombinant replication-defective human adenovirus serotype 5 (Ad5) that expresses the VP7 inner core protein of BTV serotype 8 (Ad5VP7-8) induced T-cell responses and provided protection. In the present work, we evaluated as BTV vaccine the combination of Ad5VP7-8 with another recombinant Ad5 that expresses the outer core protein VP2 from BTV-1 (Ad5VP2-1). The combination of Ad5VP2-1 and Ad5VP7-8 protected against homologous BTV challenge (BTV-1 and BTV-8) and partially against heterologous BTV-4 in a murine model. Cross-reactive anti-BTV immunoglobulin G (IgG) were detected in immunized animals, but no significant titers of neutralizing antibodies were elicited. The Ad5VP7-8 immunization induced T-cell responses that recognized all three serotypes tested in this study and primed cytotoxic T lymphocytes specific for VP7. This study further confirms that targeting antigenic determinant shared by several BTV serotypes using cellular immunity could help develop multiserotype BTV vaccines.

## Introduction

Bluetongue (BT) is a disease of compulsory notification to the OIE (World Organization for Animal Health) that affects domestic and wild ruminants and causes important economic losses (1). BT most severely affects the ovine livestock with infections in cattle and goats often being subclinical. Viremia is nonetheless present in subclinically infected animals, and this is thought to act as a reservoir for the disease (2). Bluetongue virus (BTV), the causative agent of this disease, is an arbovirus usually transmitted by the bite of *Culicoides* midges (1). BTV (family: Reoviridae; genus: *Orbivirus*) has a double-stranded RNA (dsRNA) genome virus that consists of 10 segments that encode for 7 structural proteins (VP1–VP7) and at least 4 non-structural proteins (NS1–NS4) (3–5). BT was traditionally considered as a subtropical disease with occasional incursions in Southern Europe. However, the redistribution of the competent vector *Culicoides imicola* across the Mediterranean Basin as well as the discovery that autochthonous *Culicoides* species can also harbor and transmit the virus over winter have indicated that the disease can now be considered endemic in Europe (6–9). BTV infections produce pyrexia, loss of appetite, depression, and loss of milk production in lactating animals (9). Transplacental transmission can occur even in subclinical cases, and this can lead to fetus malformation and abortions (10). The economic impact of BTV is therefore considerable and requires vaccination campaigns to keep outbreaks under control.

Vaccination, animal movement restriction, and vector population control are the main means of BTV mitigation. BTV vaccination nowadays uses inactivated virus vaccines, which in spite of their effectiveness do not provide cross-serotype protection (11, 12). Up to 28 different BTV serotypes have been reported to date (13), and serotype cross-protection is likely limited (14). This implies that in territories where multiple BTV serotypes are circulating, the livestock will require multiple immunizations to protect against each of these serotypes. Moreover, these traditional vaccines cannot differentiate infected from vaccinated animals [the so-called Differentiating Infected from Vaccinated Animals (DIVA) approach], which as a consequence restricts animal movement from affected areas toward BTV-free regions. Therefore, there is a need for the development of DIVA vaccines that could protect against multiple BTV serotypes.

Among these alternative vaccination strategies, the use of replication-defective recombinant virus vectors expressing BTV protein has shown promise in murine models and in the natural host (15, 16). The highly variable outer capsid protein VP2 contains the main antigenic determinants for neutralizing antibodies that are used to define the virus serotypes (17, 18). Although vaccination with this subunit can induce protection (19, 20), it is unlikely to induce potent cross-serotype immunity by itself. The use of more conserved BTV proteins in recombinant vaccine formulations, such as VP7 or NS1 that contains T-cell epitopes (21, 22), has already shown promise in providing some extent of cross-serotype protection (23–26). Although the immune correlates of BTV protections have to be fully defined, they are likely to depend on a combination of cellular and humoral immunity (27–30). The BTV immunity can occur in the absence of neutralizing antibodies (27, 30), which shows that induction of T-cell responses is desirable for the BTV protection. In this aspect, the BTV vaccines should aim at inducing serotype-specific neutralizing antibodies and cellular immunity to epitopes expressed in several BTV serotypes.

Adenovirus-based recombinant vaccines are good inducers of cellular immunity because high intracellular transgene expression is achieved (31). Vaccination based on the expression of immunogenic viral proteins in these recombinant vectors has shown promising results in veterinary medicine, inducing for instance protection against Peste des Petits Ruminants or foot-and-mouth disease in the natural hosts (32, 33). The use of a human adenovirus vector can also be advantageous in veterinary medicine as no previous immunity that could mitigate antigen delivery should be present in the animal host (31). We have previously reported homologous protection in the natural host with replication-defective recombinant human adenovirus serotype 5 (Ad5) that expressed VP2 and VP7 from BTV-8 [Ad5VP2-8 and Ad5VP7-8, respectively; (25)]. The partial protection was achieved in the absence of neutralizing antibodies, but a strong anti-BTV CD8^+^ T-cell response was detected upon vaccination with Ad5VP7-8. Based on this observation, we assess in this study whether vaccination with Ad5VP7-8 could be combined with immunization with other Ad5 vectors expressing VP2 from different serotypes to provide multiserotype protection. We focused our approach on BTV serotypes currently circulating in Spain and neighboring countries (BTV-1, BTV-4, and BTV-8). In the present work, we show that vaccination of interferon (IFN)-I receptor knockout mice [interferon-α/β receptor (IFNAR^−/−^)] with Ad5 vectors expressing VP2 from serotype 1 and VP7 from serotype 8 can not only protect against homologous BTV challenge (i.e., BTV-1 and BTV-8) but also provide partial protection against heterologous BTV-4 challenge.

## Materials and Methods

### Ethical Statement

All the animal experiments were carried out in a disease-secure isolation facility (BSL3) at the *Centro de Investigación en Sanidad* Animal (CISA), in strict accordance with the recommendations in the guidelines of the Code for Methods and Welfare Considerations in Behavioral Research with Animals (Directive 86/609EC; RD1201/2005), and all efforts were made to minimize suffering. Experiments were approved by the Committee on the Ethics of Animal Experiments of the Spanish *Instituto Nacional de Investigación y Tecnolog*í*a Agrar*í*a y Alimentaria* (INIA) and the National Animal Welfare Committee (PROEX 032/19).

### Cell Lines and Virus

All cell lines were cultured under standard tissue culture conditions (37°C, 5% CO_2_). Baby hamster kidney (BHK-21; ATCC CCL-10, ATCC, Manassas, VA, USA) and Vero (ATCC CCL-81) cells were grown in Dulbecco's modified Eagle's medium (DMEM), supplemented with 5% fetal bovine serum (FBS). Human Embryonic Kidney (HEK)293 cells (ATCC CRL-1573) were grown in DMEM, supplemented with 10% FBS. Mutagenized RBL-5 (T lymphoma) anti-H-2 selected variant (RMA/S) cells (kindly provided by Dr. McArdle; Nottingham Trent University, Nottingham, UK) were grown in Roswell Park Memorial Institute Medium (RPMI), supplemented with 10% FBS. The BTV stocks were obtained by the infection of BHK cells at Multiplicity of infection (MOI) 0.1 and titered in the semisolid agar medium using Vero cells as previously described (22, 34, 35). The following BTV isolates were used in the present work: BTV-1 (RSArrrr/01); BTV-4 (Spain/2004/02); and BTV-8 (NET2006/04). The BTV inactivation was performed with freshly prepared binary ethyleneimine (BEI) as previously described (22).

### Replication-Defective Adenovirus Production

Ad5DsRed and Ad5VP7-8 production has been described elsewhere (25). To generate the replication-defective adenoviruses Ad5LacZ and Ad5VP2-1, the Adeno-XSystem 3 of Clontech (Mountain View, CA, USA) was used. Briefly, the open reading frame for the *LacZ* gene and the *VP2* gene from BTV-1 were amplified by PCR and reverse transcription (RT)-PCR, respectively, and cloned with the In-Fusion HD Cloning System (Clontech) into the acceptor vector p-AdenoX-ZsGreen1 (Clontech). With the correct recombinant clones generated and linearized with PacI, HEK293 cells (providing in *trans* E1 replication function) were transfected to generate the replication-defective adenoviruses Ad5LacZ and Ad5VP2-1. Both Ad5LacZ and Ad5VP2-1 contained an expression cassette for ZsGreen1 fluorescent protein inserted in the E3 region of the adenoviral backbone. Recombinant viruses were amplified, purified, and titrated using the standard protocols and commercial kits (Clontech) as described in Martin et al. (25). Adenoviral stocks employed in the present work had the following titers: Ad5DsRed: 6 × 10^9^ IU/ml; Ad5LacZ: 6 × 10^10^ IU/ml; Ad5VP7-8: 6 × 10^9^ IU/ml; and Ad5VP2-1: 1 × 10^11^ IU/ml. Dilutions for inoculations were performed in phosphate-buffered saline (PBS).

### Polyclonal Anti-VP2-1 Sera Production

Anti-VP2-1 hyperimmune rabbit serum was obtained by two to three immunizations with Ad5VP2-1 at a 3-week interval. The presence of anti-VP2-1 immunoglobulin G (IgG) was assessed by ELISA using BTV-1 as antigen. The serum of two immunized rabbits were pooled, heat-inactivated (56°C, 30 min), and adsorbed against Vero cell lysates transferred onto polyvinylidene fluoride (PVDF) membranes. Polyclonal anti-VP2-1 rabbit specificity was confirmed in immunofluorescence studies and compared to similarly treated (heat inactivation and adsorption) sera from the same rabbits prior to immunization (pre-immune sera).

### Immunofluorescence and Confocal Microscopy

Vero cells were grown in coverslips and infected at MOI 1 with Ad5DsRed or Ad5VP2-1 for 48 h. Cells were then fixed in 4% paraformaldehyde (PFA) for 20 min at room temperature, washed in PBS, permeabilized for 10 min in PBS + 0.05% triton X-100, and unspecific binding blocked with the Dako Antibody Diluent (Dako/Agilent, Santa Clara, CA, USA) for 45 min at room temperature. Coverslips were incubated overnight at 4°C with anti-VP2-1 sera (or pre-immune sera as control) diluted in the Dako Antibody Diluent. The Alexa Fluor 647-conjugated anti-Rabbit IgG Secondary Antibody (Thermo Fisher Scientific, Waltham, MA, USA) diluted in the Dako Antibody Diluent was used to assess anti-VP2-1 antibody binding (45 min at room temperature). Nucleic acids were counterstained with 4′,6-diamidino-2-phenylindole (DAPI; Sigma-Aldrich, St. Louis, MO, USA) in PBS, and coverslips mounted in the Prolong Mounting Medium (Thermo Fisher Scientific, Waltham, MA, USA). Coverslips were counterstained with DAPI prior to mounting with Prolong Mounting Medium. The following laser lines were used for image acquisition of the fluorophores used in these experiments: DAPI 405 nm; GFP 488 nm; DsRed 561 nm; and Alexa Fluor 647 (i.e., VP2-1) 633 nm. The acquisition was performed sequentially starting from the low energy fluorophore (Alexa Fluor 647) and ending with the high energy one (DAPI). Images were captured with a ×63 objective using an LSM 880 Confocal Microscope (Zeiss, Jena, Germany). The ImageJ software (US National Institutes of Health, Bethesda, MD, USA) was used for image analysis.

### Western Blot

The Western blot analysis was performed as described in Mulens-Arias et al. (36) and Avia et al. (37). Briefly, Vero cells were mock-infected or infected with Ad5DsRed, Ad5LacZ, Ad5VP7-8, or Ad5VP2-1 at MOI 1 for 48 h. Cell lysates were obtained, resolved in sodium dodecyl sulfate polyacrylamide gel electrophoresis (SDS-PAGE), and transferred to PVDF membranes. The membranes were probed with anti-VP2-1 polyclonal sera or anti-Glyceraldehyde 3-phosphate dehydrogenase (GAPDH) antibody (#G8795; Sigma-Aldrich, St. Louis, MO, USA), antibody binding revealed with Horseradish peroxidase (HRP)-conjugated secondary anti-mouse or anti-rabbit IgG antibodies (both from GE Healthcare, Chicago, IL, USA), and protein band visualized by chemiluminescence (ECL Plus, Thermo Fisher Scientific, Waltham, MA, USA) using a ChemiDoc (Bio-Rad, Hercules, CA, USA).

### *In vivo* Experiments With Murine Model of BTV Infection

Female (7–8 weeks old) IFN-α/β receptor [IFNAR^(−/−)^] mice (38) on a C57BL/6 genetic background were housed in groups of four to six mice per cage (834 cm^2^ of floor area and 19 cm of height) in the Animal Facilities of CISA–INIA. The bedding was provided with a minimum of 2 cm of depth. Mice were immunized intramuscularly (im) twice (at 2-week interval) with 50 μl of 10^8^ infectious units (IU) of Ad5VP2-1; Ad5VP7-8; Ad5VP2-1 + Ad5VP7-8 (10^8^ IU for each construct); or Ad5DsRed + Ad5LacZ (10^8^ IU for each construct). To assess vaccination efficacy, mice were challenged subcutaneously with 10^3^ PFU of BTV-1, BTV-4, or BTV-8 two weeks after the last immunization. The power calculation was used to determine group size for these experiments (39). All groups analyzed for vaccine efficacy contained 6–12 mice. To confirm Ad5VP2-1 homologous vaccination potency, mice immunized with the four regimens as described above (four mice per group) were challenged with a lower dose of infectious BTV-1 (10^2^ PFU). The challenged mice were monitored daily for disease signs and weight loss starting at 24-h post-infection and up to day 11. Mice were euthanized to stop pain or distress according to the humane endpoints in our animal protocols. The euthanasia method used was an overdose of inhalant anesthesia (isofluorane) and cervical dislocation.

### Splenocyte Preparation and IFN-γ Enzyme-Linked Immune Absorbent Spot (ELISPOT) Assays

To assess T-cell responses, vaccinated mice from each group [Ad5DsRed + Ad5LacZ (*n* = *4*); Ad5VP2-1 (*n* = *5*); Ad5VP7-8 (*n* = *6*); and Ad5VP2-1 + Ad5VP7-8 (*n* = *7*)] were sacrificed 7 days post-booster immunization, and splenocytes prepared as described in Rojas et al. (21, 22). Splenocytes were cultured in RMPI supplemented with 10% FBS (Sigma-Aldrich, St. Louis, MO, USA) + 4 mM L-glutamine + 10 mM 4-(2-hydroxyethyl)-1-piperazineethanesulfonic acid (HEPES) + 1% × 100 non-essential amino acids + 1 mM sodium pyruvate + 100 U/ml penicillin/100 μg/ml streptomycin + 50 nM (2-mercaptoethanol) (all from Thermo Fisher Scientific, Waltham, MA, USA). Murine ELISPOT assays were performed using the MSIPS4510 Plate (Millipore Corp., Billerica, MA, USA) and as described in Rojas et al. (22). Anti-mouse IFN-γ capture antibody (clone XMG1.2, used at 10 μg/ml) and biotin anti-mouse IFN-γ detection antibody (clone R46A2, used at 5 μg/ml) (both from BioLegend, San Diego, CA, USA) were used for coating plates and detecting IFN-γ, respectively. Splenocytes (2 × 10^5^ per well) were stimulated with BEI-inactivated BTV-1, BTV-4, or BTV-8 (equivalent to 10^4^ PFU prior to inactivation/well); or mock BHK lysate as control. After 24 h incubation with the stimuli, IFN-γ spots were revealed. Concanavalin-A (1.25 μg/ml) was used as the positive control. All cultures were performed in triplicates or quadruplicates, and ELISPOT assays were considered valid only when IFN-γ spot counts in control wells were <20. An ELISPOT plate reader (AID GmbH, Strassberg, Germany) was used to quantify IFN-γ spots.

### *In vitro* Splenocyte Stimulation and Flow Cytometry-Based Cytotoxicity Assays

Splenocytes (4–5 × 10^6^/well in 24-well plates) from immunized animals were stimulated *in vitro* with VP7(283) peptide [VP7(283–291) TAILNRTTL; 10 μg/ml] for 6 days and used as effector cells in flow cytometry-based cytotoxicity assays. Syngenic RMA/S cells pulsed as previously described (40) with 10 μg/ml of relevant peptide VP7(283) or irrelevant peptide NS1(152) [NS1(152–160) GQIVNPTFI] from BTV [known to elicit T-cell responses in IFNAR^(−/−)^ mice (22, 26)] were used as target cells. Target cells were labeled with PKH67 dye as described in Rojas et al. (41), and cytotoxicity was performed as previously described in Rojas et al. (42). The gating strategy has been described in Rojas et al. (42, 43). The target cell death was evaluated with propidium iodide (PI) staining. Spontaneous and maximum cell death (measured by addition of 0.2% saponin) was included for all target cells. Specific cell lysis was measured using the following formula: % specific lysis = (% PI^+^ target cells – % spontaneous target cell death)/(% maximum target cell death – % spontaneous target cell death) ×100. Samples were acquired on a FACScalibur flow cytometer (BDbiosciences, San José, CA, USA), and the analysis was performed using the FlowJo software (BDbiosciences, San José, CA, USA).

### Serum Preparation and Detection of Anti-BTV IgG in Immunized Mice by ELISA

Sera from immunized mice were obtained prior to immunization (day 0) and 10 days after booster immunization. Sera from mice that survived the infectious BTV challenge were obtained on day 15 post-challenge. All sera were heat inactivated (56°C, 30 min) prior to use in ELISA or seroneutralization assays. ELISA for anti-BTV IgG were based on previous reports (33). Briefly, ELISA plates (Nunc MaxiSorp, Thermo Fisher Scientific, Waltham, MA, USA) were coated with the equivalent to 10^4^ PFU/well of BTV-1, BTV-4, or BTV-8 in PBS overnight at 4°C. Plates were then washed with PBS–T (PBS + 0.1% Tween), blocked with PBS–T + 5% skimmed milk for 1 h at room temperature, and serial serum dilutions (in PBS-T + 2% skimmed milk) were incubated for 2 h at room temperature. Plated were then washed in PBS–T, and IgG binding was detected with an HRP-conjugated anti-mouse IgG secondary antibody (Bethyl Laboratories, Inc., Montgomery, TX, USA) diluted in PBS–T + 2% skimmed milk and revealed with 3,3′,5,5′-Tetramethylbenzidine (TMB) substrate (Thermo Fisher Scientific, Waltham, MA, USA) after extensive washing. The optical density was read at 450 nm (OD450 nm) on a FluoSTAR Omega ELISA plate reader (BMG Labtech, Ortenberg, Germany) after stopping the colorimetric reaction with 3N sulfuric acid. IgG titer are expressed as the reciprocal value using a linear regression of the serum dilutions for which OD450 nm reading in immune serum dilution reaches two times that of the OD450 nm reading in the pre-immune serum of the same animal as previously described (33). The negative dilution values were given a value of 1 for the graphical representation.

### Bluetongue Virus Seroneutralization Assays

The BTV seroneutralization assays were performed as previously described (25, 44). Briefly, BTV serotypes 1, 4, or 8 (of 100 PFU) were incubated with serial serum dilutions (starting at 1:50 and up to 1:800) for 1 h at 37°C in 96-well plates. Vero cells (2 × 10^4^/well) were then added and cultured for 4–5 days until complete cytopathic effect (CPE) was detected in the negative control wells. Cells were then fixed with 2% paraformaldehyde and visualized by crystal violet staining. Seroneutralization titers were calculated as the reciprocal serum dilution at which <50% of CPE was observed in the Vero cells.

### Statistical Analysis

A statistical analysis was performed using the Graphpad Prism Software (San Diego, CA, USA). Statistical tests used to evaluate significance in experiments are mentioned in the figure legends. Levels of significance were defined as ^*^p < 0.05, ^**^p < 0.01, and ^***^p < 0.001.

## Results

### Construction of a Recombinant Adenovirus that Expresses the Subunit VP2 From BTV Serotype 1 (Ad5VP2-1)

**Figure 1:**
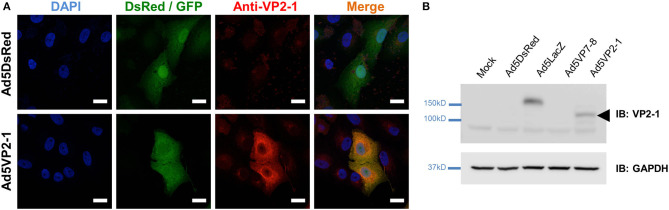
VP2-1 protein is expressed in Ad5VP2-1-infected Vero cells. **(A)** Representative immunofluorescence confocal images of Vero cells infected with Ad5DsRed or Ad5VP2-1 (MOI 1) and stained with anti-VP2-1 polyclonal rabbit sera (shown in red). Reporter fluorescent proteins expressed by the Ad5DsRed (DsRed) or Ad5VP2-1 (GFP) constructs are shown in green. 4′,6-Diamidino-2-phenylindole (DAPI) was used to counterstain for nucleic acids (blue). Scale bar = 20 μm. **(B)** Western blot of Vero cells mock-infected or infected with Ad5DsRed, Ad5LacZ, Ad5VP7-8, or Ad5VP2-1. PVDF membranes were immunoprobed (IB) with anti-VP2-1 polyclonal rabbit sera or anti-GAPDH antibody (as sample loading control). Arrowhead indicates the presence of VP2-1 in the Ad5VP2-1-infected cells at the predicted molecular weight of the protein.

An Ad5 expressing the subunit VP2 from the outer viral capsid of BTV-1 (Ad5VP2-1) was produced to evaluate its potency as BTV vaccine. The VP2 ORF from BTV-1 was amplified by RT-PCR from the viral RNA extract and cloned into p-AdenoX-ZsGreen1 by the InFusion Clontech System (see section Materials and Methods). Insert presence was confirmed by the restriction enzyme digestion, sequencing, and RT-PCR. To detect VP2-1 protein expression in cells, we produced a polyclonal rabbit serum by utilizing the pooled hyperimmune sera from two rabbits immunized with Ad5VP2-1. The anti-VP2-1 immune serum could recognize Ad5VP2-1-infected cells, whereas the pre-immune serum did not bind to these cells (Supplementary Figure 1), demonstrating the specificity of the immune serum. Immunofluorescence studies confirmed the expression of VP2-1 in Ad5VP2-1-infected cells (Figure 1A). The VP2-1 expression was detected in Ad5VP2-1-infected Vero cells but not in control Ad5LacZ/Ad5DsRed-infected cells. The Western blot analysis confirmed the expression of VP2-1 in Vero cell lysates from Ad5VP2-1-infected cells (Figure 1B). A protein band at the predicted molecular weight for VP2 was detected in Ad5VP2-1-infected cells but not in mock-infected cells or Ad5DsRed-, Ad5LacZ-, or Ad5VP7-8-infected cells. These data demonstrate that the VP2-1 protein is expressed in Ad5VP2-1-infected cells, which indicate that Ad5VP2-1 could be used for vaccination studies. The generation of the other recombinant adenoviruses used in the present work has been reported elsewhere (25, 45).

### Vaccination With Ad5VP2-1 and Ad5VP7-8 Provides Protection Against BTV-1, BTV-4, and BTV-8 Challenges

**Figure 2:**
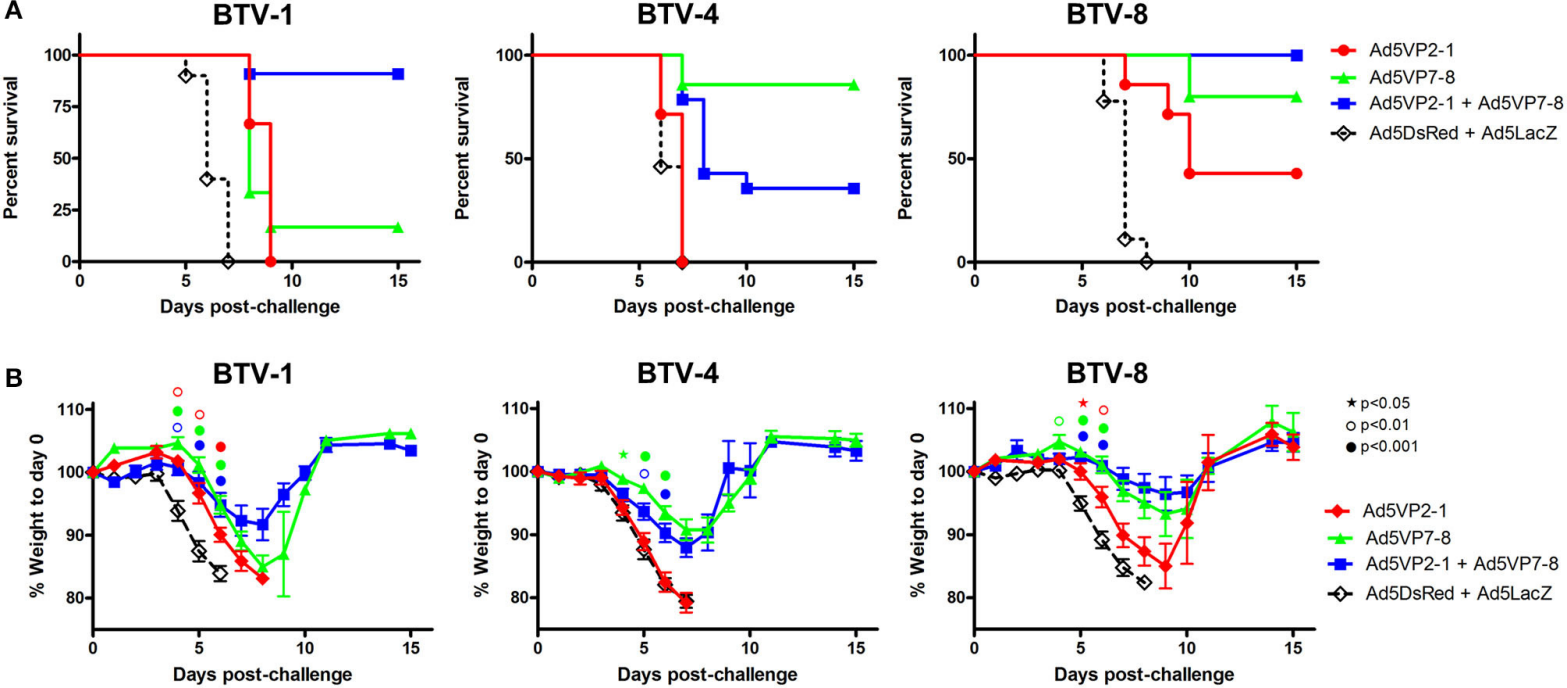
The Ad5VP2-1 and Ad5VP7-8 immunization confers protection against autologous and heterologous BTV challenges. IFNAR^(−/−)^ mice were immunized with Ad5VP2-1, Ad5VP7-8, Ad5VP2-1 + AdV5P7-8, or with Ad5DsRed + Ad5LacZ as control and challenged with 1,000 PFU of BTV-1, BTV-4, or BTV-8. **(A)** Survival plots for each immunization group after BTV challenge (serotype indicated above survival graphs). **(B)** Mouse weight post-challenge (mean % to prechallenge weight ± SEM) are plotted for each immunized group after BTV serotype challenge (indicated above graphs). Statistical comparison was performed up to day 6 with an intact group of animals. ^⋆^*p* < 0.05; **°***p* < 0.01; ^•^*p* < 0.001; symbol color refers to immunization group (red, Ad5VP2-1; green, Ad5VP7-8; and blue, Ad5VP2-1 + Ad5VP7-8); one-way ANOVA with Tukey post-test (Ad5VP2-1; Ad5VP7-8; or Ad5VP2-1 + Ad5VP7-8 vs. Ad5DsRed + Ad5LacZ).

Previous work from our group has indicated that vaccination with a combination of recombinant replication-defective adenoviruses expressing the highly variable outer capsid protein VP2 and the more conserved inner capsid protein VP7 from BTV-8 protected against homologous virus challenge in mice and sheep (25). We wanted to assess in a murine model of BTV infection whether combining the immunization against VP2 from a serotype (in the present work serotype 1) with the immunization against VP7 from a different serotype (in the present work serotype 8) could protect against homologous (i.e., BTV-1 and BTV-8) and heterologous (i.e., BTV-4) BTV challenges. We, therefore, vaccinated IFNAR^(−/−)^ mice with Ad5VP2-1 alone, Ad5VP7-8 alone; a combination of Ad5VP2-1 and Ad5VP7-8; or, as control, a combination of Ad5DsRed and Ad5LacZ and challenged each vaccination group with BTV-1, BTV-4, or BTV-8 (Figure 2). The combination of Ad5VP2-1 and Ad5VP7-8 vaccination conferred protection against BTV-1 challenge (Figure 2A) with >90% survival rates. The vaccination with Ad5VP2-1 only produced a delay in disease onset against BTV-1. The Ad5VP7-8 vaccination delayed disease onset (an 8-day median survival for the Ad5VP7-8 vaccine group vs. a 6-day median survival for the Ad5DsRed + Ad5LacZ group) and provided very limited protection (≈17% survival) against BTV-1. Since the BTV infection in IFNAR^(−/−)^ mice leads to loss of appetite, we weighed the mice daily as a surrogate for the disease progression (Figure 2B). Weight loss data confirmed that the Ad5VP2-1 and Ad5VP7-8 vaccination delayed the BTV-1 disease onset. The combination of both adenovirus vaccines further reduced the weight loss but did not completely abolish it, indicating that IFNAR^(−/−)^ mice still developed the disease. To evaluate whether the vaccination with Ad5VP2-1 alone could protect against the BTV-1 infection, we challenged the mice with 10^2^ PFU BTV-1 instead of 10^3^ PFU used in the previous experiments (Supplementary Figure 2). The Ad5VP2-1 vaccination could protect 50% of mice with this lower dose of BTV-1 challenge showing that Ad5VP2-1 can elicit protective immunity. The Ad5VP7-8 immunization could protect 100% of mice against this dose of BTV-1 challenge.

The aim of the work presented here was also to evaluate the protective potency across several BTV serotypes of combining the vaccination of Ad5 vectors that express the serotype variable VP2 with an Ad5 vector that expresses the more conserved VP7, which is a source of T-cell epitopes shared by multiple BTV serotypes (21, 46). We, therefore, evaluated protection in vaccinated mice against BTV-8, which expresses a heterologous VP2 but a homologous VP7 (Figure 2). As previously reported (25), Ad5VP7-8 vaccination protected against BTV-8 challenge. The addition of Ad5VP2-1 appears also to slightly improve protection against BTV-8. Vaccination with Ad5VP2-1 alone also provided partial protection against BTV-8, indicating that partial cross-serotype protection could be achieved with this adenoviral construct (Figure 2A). Weight loss data confirmed BTV-8 survival data (Figure 2B). The Ad5VP2-1 vaccination thus appears to offer some protection against BTV-8 challenge and combined with Ad5VP7-8 conferred 100% survival rates.

We also assessed protection against the challenge with the heterologous serotype BTV-4. As predicted, the Ad5VP2-1 vaccination did not protect against BTV-4 challenge. Approximately 40% of mice vaccinated with a combination of Ad5VP2-1 and Ad5VP7-8 survived BTV-4 challenge, whereas vaccination with Ad5VP7-8 alone resulted in ≈85% survival rate (Figure 2A). Weight loss data confirmed BTV-4 survival data (Figure 2B). Thus, it appears that the Ad5VP7-8 vaccination provides substantial partial protection against the challenge with heterologous BTV-4.

### The Ad5VP2-1 and Ad5VP7-8 Immunization Produces Cross-Reactive Anti-BTV IgG but Fails to Elicit Seroneutralizing Antibodies

**Figure 3:**
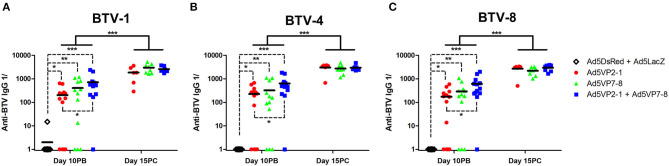
The Ad5VP2-1 and Ad5VP7-8 immunization produces anti-BTV IgG that recognize heterologous BTV serotypes. The presence of anti-BTV IgG specific for **(A)** BTV-1, **(B)** BTV-4, and **(C)** BTV-8 in the sera of immunized mice was assessed by ELISA at day 10 post-boost (day 10PB; *n* = 12–14/group) and at day 15 post-challenge in surviving mice (day 15PC; *n* = 6–8/group). Data are presented as the reciprocal dilution (anti-BTV IgG 1/) necessary to achieve 2-fold absorbance for the naïve serum. ^*^p < 0.05; ^**^p < 0.01; ^***^p < 0.001 [one-way ANOVA with Dunn's post-test (Ad5DsRed + Ad5LacZ vs. Ad5VP2-1; Ad5VP7-8; or Ad5VP2-1 + Ad5VP7-8); unpaired *t*-tests (day 10PB vs. day 15PC in Ad5VP2-1; Ad5VP7-8; and Ad5VP2-1 + Ad5VP7-8 groups)].

We next wanted to identify some of the immune factors induced by the vaccination that correlated with BTV protection in this murine model. The presence of IgG specific for the different BTV serotypes used for *in vivo* challenge was assessed using the sera of immunized animals (Figure 3). The Ad5VP2-1 and AdVP7-8 immunization elicited the production of IgG that could recognize all three BTV serotypes. The combination of Ad5VP2-1 and Ad5VP7-8 appears to improve the amount of anti-BTV IgG present in the sera of immunized mice. The amount of cross-reactive anti-BTV IgG detected in the sera of surviving mice increased after the BTV challenge. The presence of anti-BTV IgG capable of recognizing all three BTV serotypes after vaccination could therefore represent one of the immunological bases through which cross-serotype protection is occurring *in vivo*.

**Figure 4:**
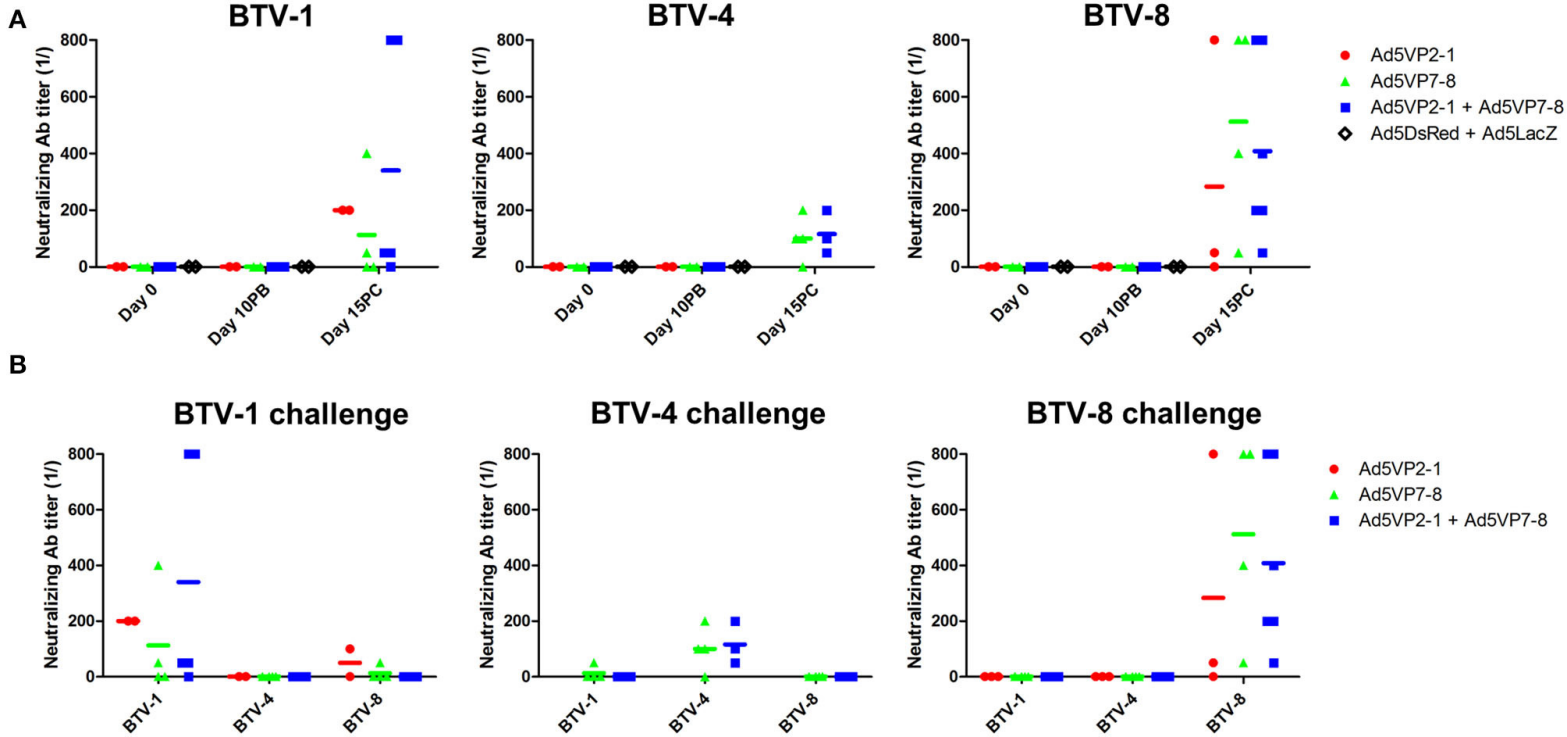
The Ad5VP2-1 and Ad5VP7-8 immunization does not produce significant levels of BTV-neutralizing antibodies. **(A)** BTV-1,−4, or−8 (100 PFU) preincubated with diluted sera were used to infect Vero cells to assess the presence of BTV neutralizing antibodies. Sera obtained prior to immunization (day 0) at 10 days after booster vaccination (day 10PB) and at 15 days after BTV challenge (day 15PC) in surviving mice (*n* = 2–6/group) were used in these experiments. Data are presented as the reciprocal dilution at which <50% complete cytopathic effect (CPE) was observed. **(B)** The presence of neutralizing antibodies cross-reactive to other BTV serotypes in the sera of mice that survived the challenged was assessed using day 15 post-challenge sera. The serotype specified above the plot indicates the challenge received by the mice, while the x-axis shows the BTV serotype used for seroneutralization.

The presence of neutralizing antibodies is nonetheless generally accepted as a better correlate with protection in BTV than anti-BTV IgG presence. We, therefore, assessed the presence of neutralizing antibodies in the sera of immunized mice (Figure 4A). None of the vaccination regimens induced significant levels of neutralizing antibodies at day 10 post-booster immunization. Indeed, neutralizing antibodies were only detected in the mice that survived the challenge. Remarkably, these neutralizing antibodies elicited during BTV challenge only inhibited BTV infection in the serotype that was used for the *in vivo* challenge (Figure 4B) and thus showed little to no cross-reaction between serotypes. This indicates that neutralizing antibody responses are mostly triggered after the challenge and are therefore unlikely to explain the cross-protection observed in the *in vivo* experiment.

### The Ad5VP2-1 and Ad5VP7-8 Immunization Induces T-Cell Responses Against BTV-1, BTV-4, and BTV-8 and Activates Anti-VP7 Cytotoxic T Lymphocytes

**Figure 5:**
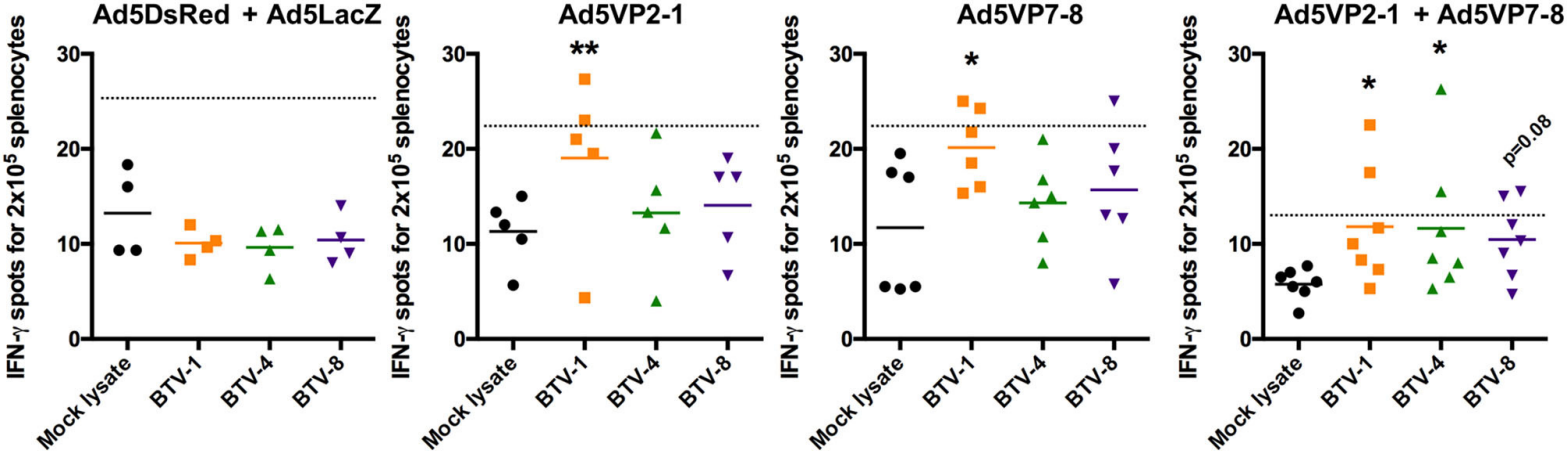
The Ad5VP2-1 and Ad5VP7-8 immunization induces T-cell responses against homologous and heterologous BTV serotypes. IFN-γ ELISPOT assays of splenocytes from Ad5VP2-1, Ad5VP7-8, Ad5VP2-1 + Ad5VP7-8, or Ad5DsRed + Ad5LacZ immunized IFNAR^(−/−)^ mice cultured with inactivated BTV-1,−4, or−8, or mock lysate as control. Mean IFN-γ spots for 2 × 10^5^ splenocytes obtained from immunized mice are presented. ^*^p < 0.05; ^**^p < 0.01; one-way ANOVA with Dunnett's post-test (BTV-1, BTV-4, or BTV-8 vs. mock lysate). Dotted line indicates twice the mean of IFN-γ spots in mock lysate stimulated splenocytes.

Based on the observation that T-cell responses could provide at least partial protection against heterologous BTV challenge (28), and that adenoviral vectors are excellent at priming T-cell responses against their transgene (31), we aimed at evaluating the cellular response induced by our vaccination regimen. We first assessed IFN-γ production against BEI-inactivated BTV-1, BTV-4, and BTV-8 in splenocytes obtained from immunized mice after the booster vaccination (Figure 5). No specific IFN-γ production in BTV-1, BTV-4, or BTV-8 was detected in the control group (Ad5DsRed + Ad5LacZ). The Ad5VP2-1 immunization induced the activation of IFN-γ producing splenocytes against BTV-1, but not BTV-4 or BTV-8. The Ad5VP2-1 immunization thus appears to elicit T-cell responses mostly against BTV-1. A specific IFN-γ response to BTV-1 was also detected in splenocytes obtained from Ad5VP7-8-immunized mice. Finally, the combination of Ad5VP2-1 and Ad5VP7-8 vaccination-induced IFN-γ responses against BTV-1, BTV-4, and BTV-8. It thus appears that vaccination with Ad5VP2-1 and particularly Ad5VP7-8 can elicit T-cell responses that recognize multiple BTV serotypes.

**Figure 6:**
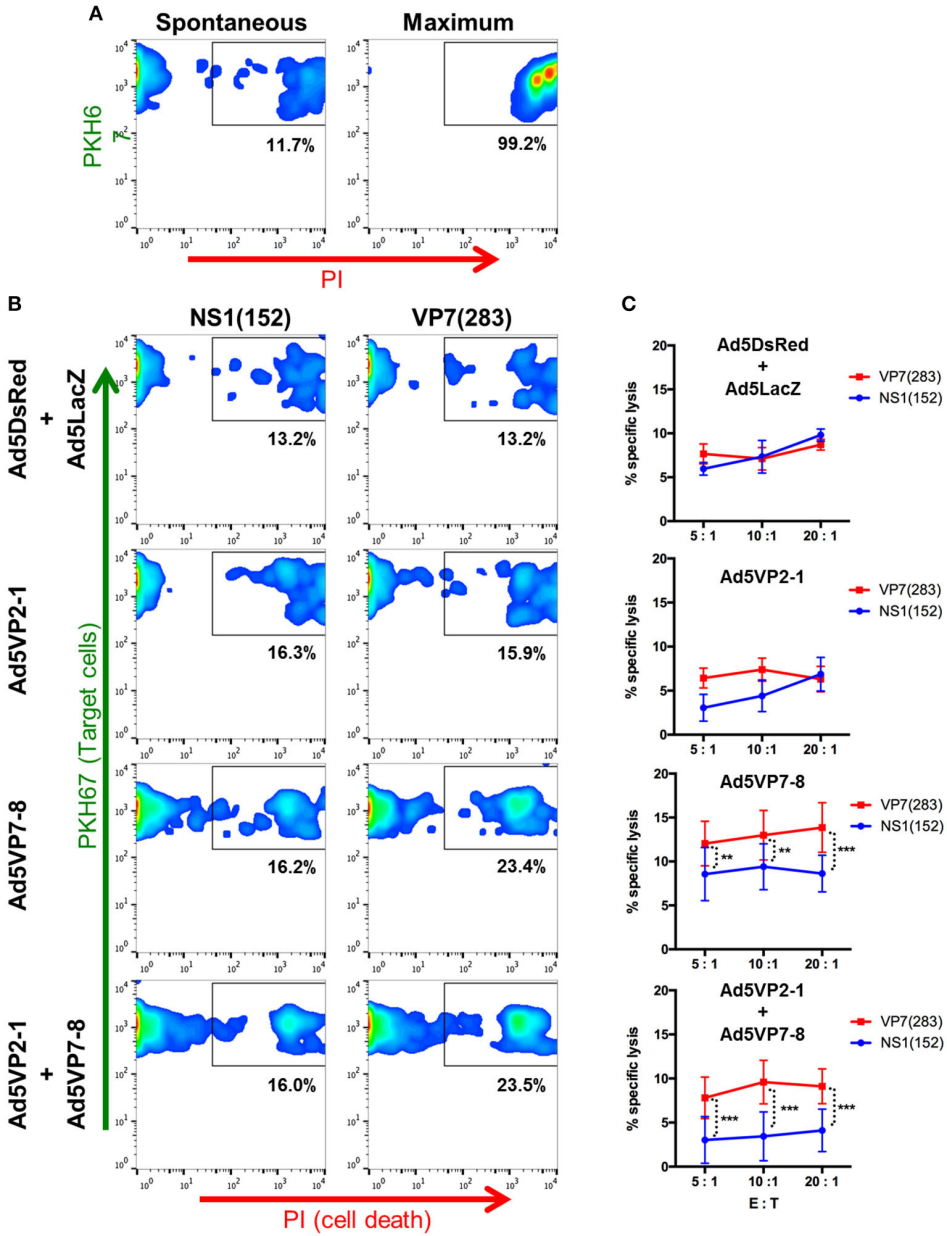
The Ad5VP7-8 immunization induces cytotoxic T lymphocytes (CTL) against the VP7(283) peptide. Splenocytes from immunized mice were restimulated *in vitro* with the VP7(283) peptide and used as effector cells in flow cytometry-based cytotoxicity assays. RMA/S cells pulsed with VP7(283) peptide or irrelevant peptide NS1(152) were labeled with PKH67 dye and used at target cells. Propidium iodide (PI) staining was used to evaluate cell death. **(A)** Dot plot examples of spontaneous and maximum target cell death. **(B)** Dot plot examples of peptide-pulsed RMA/S target cell death in co-cultures with splenocytes obtained from Ad5DsRed + Ad5LacZ, Ad5VP2-1, Ad5VP7-8, or Ad5VP2-1 + Ad5VP7-8 immunized mice. **(C)** Percentage (mean ± SD) of target cell specific lysis induced by splenocytes from Ad5DsRed + Ad5LacZ (*n* = *3*), Ad5VP2-1 (*n* = 4), Ad5VP7-8 (*n* = 6), or Ad5VP2-1 + Ad5VP7-8 (*n* = 5) immunized mice at different effector to target cell ratios (E:T). ^**^p < 0.01; ^***^p < 0.001; two-way ANOVA with Sidak's post-test [NS1(152) vs. VP7(283)].

To further confirm the existence of effector T-cell mechanisms capable of targeting multiple BTV serotypes, we assessed the presence of anti-BTV cytotoxic T lymphocytes (CTL) in vaccinated mice (Figure 6). Several BTV-specific T-cell epitopes for VP7 and NS1 have been described in IFNAR^(−/−)^ mice (21, 22, 26), since these viral proteins are among the most conserved in BTV. We chose the VP7(283) peptide to assess CTL responses induced by Ad5VP7-8 vaccination as its sequence is conserved in all three serotypes herein used. The peptide NS1(152), known to also elicit CTL responses (22, 26), was chosen as an irrelevant peptide in this context. The Ad5VP7-8 vaccination primed CTL capable of specifically lysing target cells pulsed with VP7(283) peptide (Figure 6). Similarly, the combination of Ad5VP2-1 and Ad5VP7-8 also primed CTL against VP7(283). Vaccination with Ad5VP2-1 alone or the combination of Ad5DsRed and Ad5LacZ was not capable of producing anti-VP7(283) CTL responses. Overall, it appears that the Ad5VP7-8 vaccination can elicit anti-BTV CTL responses against peptides that are conserved across the three serotypes used in the present work. These cells could therefore be one of the effector mechanisms that confer protection across multiple BTV serotypes.

## Discussion

In the present work, we aimed to evaluate the potential of replication-defective adenoviral vectors that express BTV variable outer capsid protein VP2 and the more conserved inner capsid protein VP7 as a vaccine against multiple BTV serotypes. Previous work showed that this combination of adenovirus-based vaccines protected against homologous virus challenge in a murine model and in the natural host (25). In this work, we showed that a heterologous formulation (i.e., the delivery of VP2 from one serotype with VP7 from a different serotype) can provide protection against several serotypes. This combination of adenoviral vectors protected against the challenge from the BTV serotypes used to produce the adenoviral vaccine vectors, and more intriguingly against a heterologous BTV challenge.

Developing BTV vaccines capable of providing multiserotype protection is one of the major milestones to achieve in the field of BTV vaccination. Currently, most vaccination, and by extension control strategies for BTV in the field, rely on inactivated virus vaccines that only protect against a specific BTV serotype. This implies that when a new serotype starts to circulate in a territory, a new vaccination campaign is necessary to provide protection for the livestock. Our data indicate that delivering BTV capsid proteins through an adenoviral vector could help elicit broader immunity to BTV than conventional vaccines. Moreover, the adenoviral platform described here could be implemented as a DIVA vaccine. Indeed, we show that vaccination with Ad5VP7-8 elicited CTL responses to the immunogenic peptide VP7(283) but not NS1(152), an immunodominant CTL epitope from a BTV non-structural protein (26). This implies that Ad5VP2-1 and Ad5VP7-8 could be considered DIVA vaccines after establishing DIVA tests that differentiate immunity against structural BTV proteins, such as VP2 or VP7, and from immunity against non-structural proteins, such as NS1, which would only be triggered after a viral infection.

In spite of inoculating all the mice with the same amounts of PFU (10^3^) for each serotype, differences in infection kinetics were observed. For instance, in control Ad5DsRed + Ad5LacZ groups, weight loss started at day 4 post-challenge for BTV-1 and BTV-4 and at day 5 post-challenge for BTV-8. BTV-1-infected control mice also displayed a more severe respiratory disease than with BTV-4 or BTV-8, which reached endpoint criteria prior to the >20% weight loss endpoint. Differences in strain virulence are frequent in the BTV infection and are multifactorial (47–49). For instance, the BTV-8 strain responsible for the 2006 outbreak in northern Europe showed increased clinical signs in cattle when compared to other BTV serotypes (50). Differences in infection kinetics are probably due to the difference in virulence of the three serotypes used in the present study.

The combination of Ad5VP2-1 and Ad5VP7-8 provided partial protection against heterologous challenges. Our data indicate that this was mostly due to the immunity to VP7 induced by the vaccine. More specifically, we detected the specific production of IFN-γ against BTV antigens and the generation of CTL against a VP7 immunogenic peptide in splenocytes from the AdVP7-8-immunized mice. Vaccination with Ad5VP2-1 and Ad5VP7-8 also generated IgG capable of recognizing different BTV serotypes, but no significant BTV-neutralizing antibody titers were detected after immunization. The presence of IgG specific for the more conserved VP7 is often used in diagnostics to identify the BTV seroconversion in herds, while neutralizing antibodies to the highly variable VP2 are employed to define BTV serotypes (9). The presence of cross-reactive IgG was not only seen in vaccine regimen that included Ad5VP7-8, but also in Ad5VP2-1-vaccinated mice. Thus, the Ad5VP2-1 immunization appears to induce cross-reactive IgG that recognize multiple BTV serotypes but are unable to neutralize infection. It is possible that these Igs-G are raised against conserved regions of VP2 among serotypes that are not neutralizing determinants but could still affect viral replication. This could contribute to the heterologous protective effects against BTV-8 that Ad5VP2-1 immunization provided. The immunological relevance of this finding still remains unclear, and further work will be required to better evaluate the role of these anti-BTV IgGs directed to VP2. Overall, it appears that triggering cellular immunity against VP7 would be useful to provide immunity against multiple serotypes in a similar way as immunity to NS1 has been described to provide multiserotype protection in IFNAR^(−/−)^ mice (26). Indeed, sequence alignment of VP7 from the BTV strains used in the present work showed the high degree of conservation of the protein between serotypes (Supplementary Figure 3). Effective VP7 delivery as a vaccine is however unlikely to be sufficient to induce full heterologous protection as illustrated by Bouet-Cararo et al. (51). They found that VP7 immunization using a replicative-defective canine adenovirus only provided partial homologous protection and not heterologous protection in sheep. The combination of VP7 with other BTV proteins will probably improve vaccine efficacy. For instance, a DNA-recombinant modified vaccinia virus Ankara (rMVA) prime boost strategy using BTV-4 VP2, VP7, and NS1 expression led to heterologous protection against BTV-1 and BTV-8 in IFNAR^(−/−)^ mice (52). However, in this system, the combination of VP2 and VP7 expression showed only around 30% protection against homologous challenges. NS1 inclusion in the vaccination regime however boosted protection to up to 100%. This BTV antigen combination was also successful in inducing heterologous BTV protection using avian reovirus microspheres as a delivery system (19). Therefore, understanding the minimal BTV antigen requirements for multiserotype protection remains elusive, but from our work and others, it appears that these formulations should probably include VP7. We also detected cellular immunity to BTV in Ad5VP2-1-immunized mice, which could contribute to immunity against BTV. The combination of Ad5VP2-1 and Ad5VP7-8 vaccination appears to improve therapy in the cases of BTV-1 and BTV-8 challenge when compared to vaccination with either adenovirus alone. However, in the case of BTV-4 challenge, it appears that this combination has a negative impact on the therapeutic effects provided by the Ad5VP7-8 vaccine. Indeed, the Ad5VP2-1 vaccination had no protective effects on the BTV-4 infection. The VP2 protein sequence alignment between the three serotypes showed a greater homology between VP2 proteins from BTV-1 and BTV-8 than from BTV-4 (Supplementary Figure 3). It could be that the cellular immunity induced by Ad5VP2-1 in the case of BTV-4 challenge “dilutes” the cellular immunity provided by Ad5VP7-8 due to the presence of immunodominant T-cell epitopes on VP2. Immunodominance is a multifactorial immune mechanism designed to fine tune T-cell responses toward more abundant and/or better presented epitopes (53, 54). During T-cell responses, antigen-specific T cells compete for growth factors, a mechanism that favors the expansion of T cells that receive a strong signaling through their TCR in detriment of T cells that only received moderate activation (55–58). This can in some cases limit T-cell response efficacy, as it can narrow the spectrum of T cells responding to a particular antigen. This is predictably more likely to occur if T cells specific for two different antigens are simultaneously competing for the same growth factors. T-cell response induced by the Ad5VP2-1 immunization could thus be limiting the T-cell responses to VP7 in our experimental setting. In the case of BTV-1 and BTV-8 challenge, this had no detrimental effects on protection as Ad5VP2-1 vaccination had protective effects by itself, but in the case of BTV-4 heterologous challenge, AdVP7-8 therapeutic efficacy appears diminished due to responses to Ad5VP2-1. This is an important consideration for the vaccination regimen that could be for instance solved by alternating antigen vaccination so that minimal epitope competition occurs.

As for most antiviral immune responses, protection against BTV is likely to depend on a combination of cellular and humoral immunity. Cellular and humoral components of immunity have proved important for BTV protection in the natural host (28, 29), though protection can occur in the absence of neutralizing antibodies (27, 30). Adenovirus-based recombinant vaccines are good inducers of cellular immunity (31); however, they may not be optimal for the induction of humoral responses in our experimental setting. Our data indicate that in spite of inducing IgG that recognize all BTV serotypes, Ad5VP2-1 did not produce significant titers of BTV-neutralizing antibodies. Neutralizing antibodies were nonetheless detected in the mice that survived BTV challenge, indicating that these could be important for protection in this murine model. Altering the route through which the antigen is detected by the immune system could be studied to improve neutralizing antibody production with adenovirus-based vaccine. Soluble forms of antigens are more likely to give rise to antibody responses, as exemplified by inactivated virus vaccines that often trigger good antibody responses but are usually poor inducers of cellular immunity due to the lack of intracellular replication (59–61). Delivery of soluble proteins by adenoviral vector is feasible (62–64) and could be implemented to promote humoral immunity to a soluble antigen. Overall, it appears that understanding the immune correlates with disease protection is critical to improve vaccine design in BTV and in other infectious diseases.

We herein describe an adenovirus-based vaccination approach for BTV that provides multiserotype protection. Adenoviral vectors have multiple benefits as alternative vaccination strategies. They are relatively easy to manipulate, and high titers of stable vaccine can be obtained. They are effective at antigen delivery and provide an adjuvant effect that promotes long-lasting adaptive immunity. Moreover, their safety for veterinary use has been demonstrated (65), and an adenovirus-based vaccine for the foot-and-mouth disease has been approved in the USA in cases of emergency. The combination of adenovirus vectors that express VP2 and VP7 capsid proteins from BTV could therefore represent an interesting strategy to improve the cross-reactive immunity against BTV. The Ad5VP7-8 construct appears particularly promising for providing a baseline protective immunity across BTV serotypes. Although much work remains to optimize vaccination regimens, the present study highlights the immunogenic potential of adenovirus-based strategies for the development of DIVA vaccines in BTV.

## Data Availability Statement

The raw data supporting the conclusions of this article will be made available by the authors, without undue reservation.

## Ethics Statement

The animal study was reviewed and approved by the Committee on the Ethics of Animal Experiments of the Spanish Instituto Nacional de Investigación y Tecnología Agraría y Alimentaria (INIA) and the National Animal Welfare Committee (PROEX 032/19).

## Author Contributions

JR, VM, and NS designed the experiments and directed the work. JR, DB-M, MA, and VM performed the experiments. JR and VM wrote the manuscript. All authors contributed to the article and approved the submitted version.

## Conflict of Interest

The authors declare that the research was conducted in the absence of any commercial or financial relationships that could be construed as a potential conflict of interest.
