# Supplementary figures and images for: Vaccination With Recombinant Adenoviruses Expressing the Bluetongue Virus Subunits VP7 and VP2 Provides Protection Against Heterologous Virus Challenge
Source: Front Vet Sci. 2021 Mar 10;8:645561. doi: 10.3389/fvets.2021.645561 (PMC7987666; doi:10.3389/fvets.2021.645561)

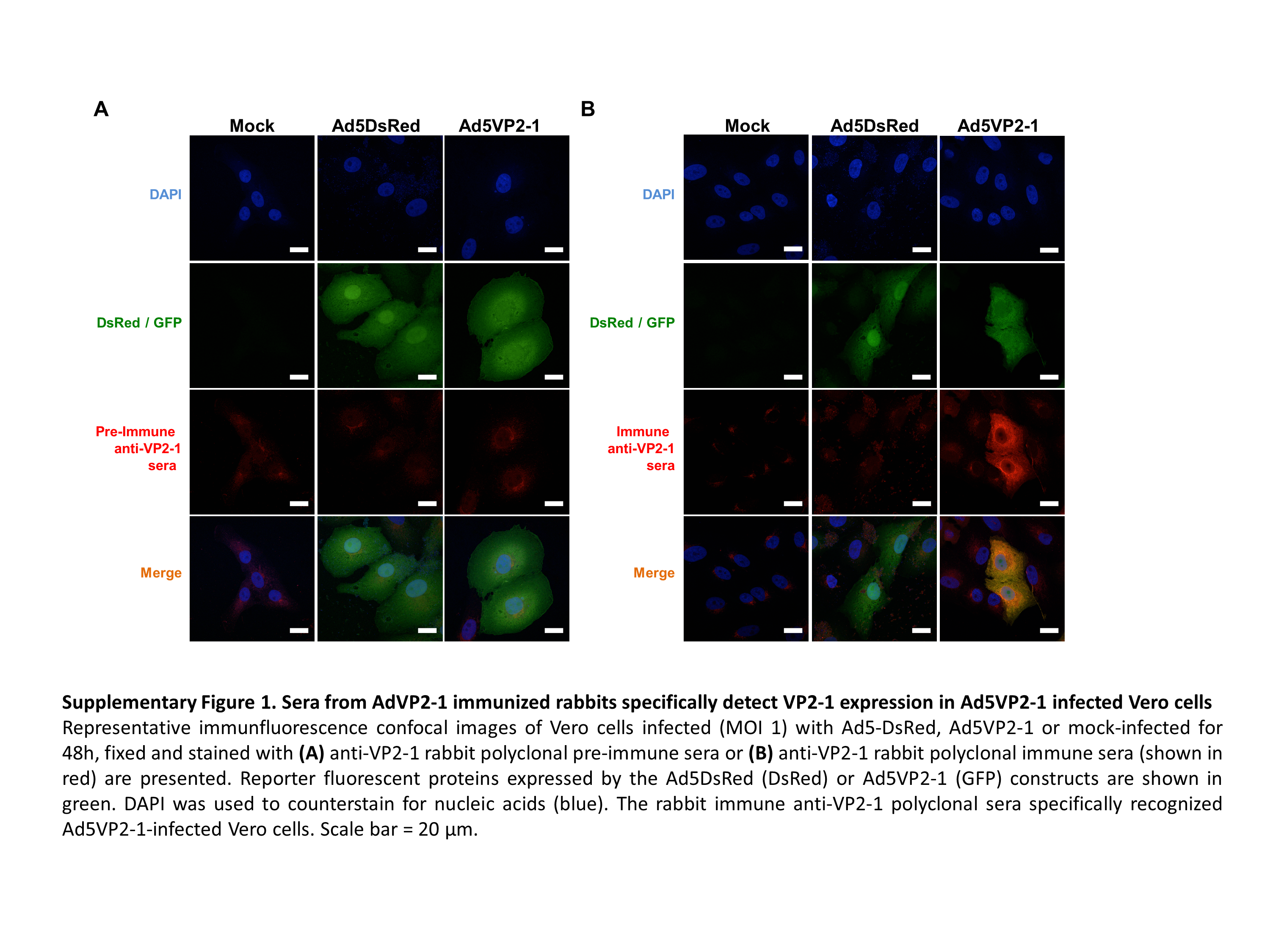

Supplement: Supplementary file 1 [file Image_1.TIF]

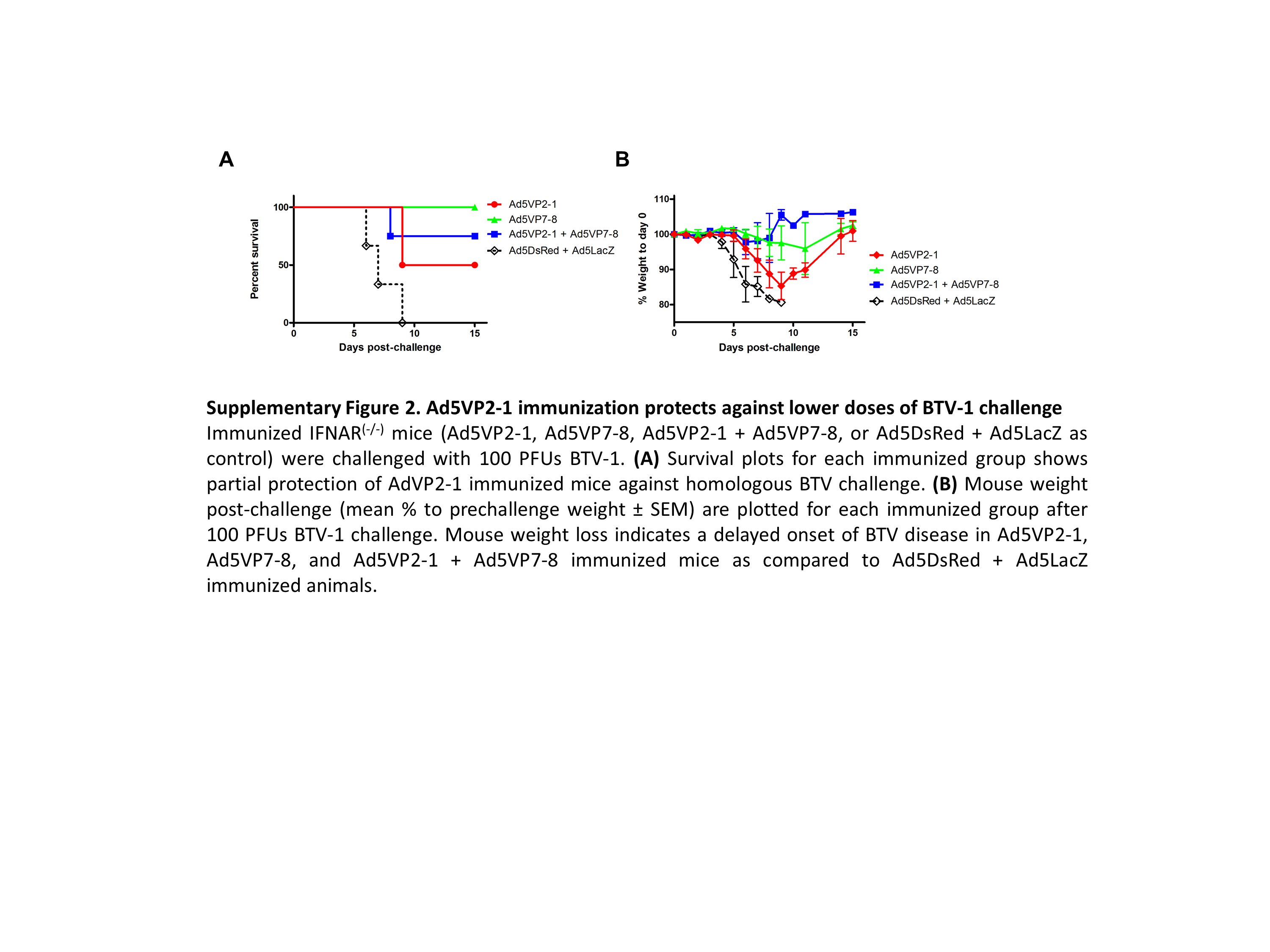

Supplement: Supplementary file 2 [file Image_2.TIF]

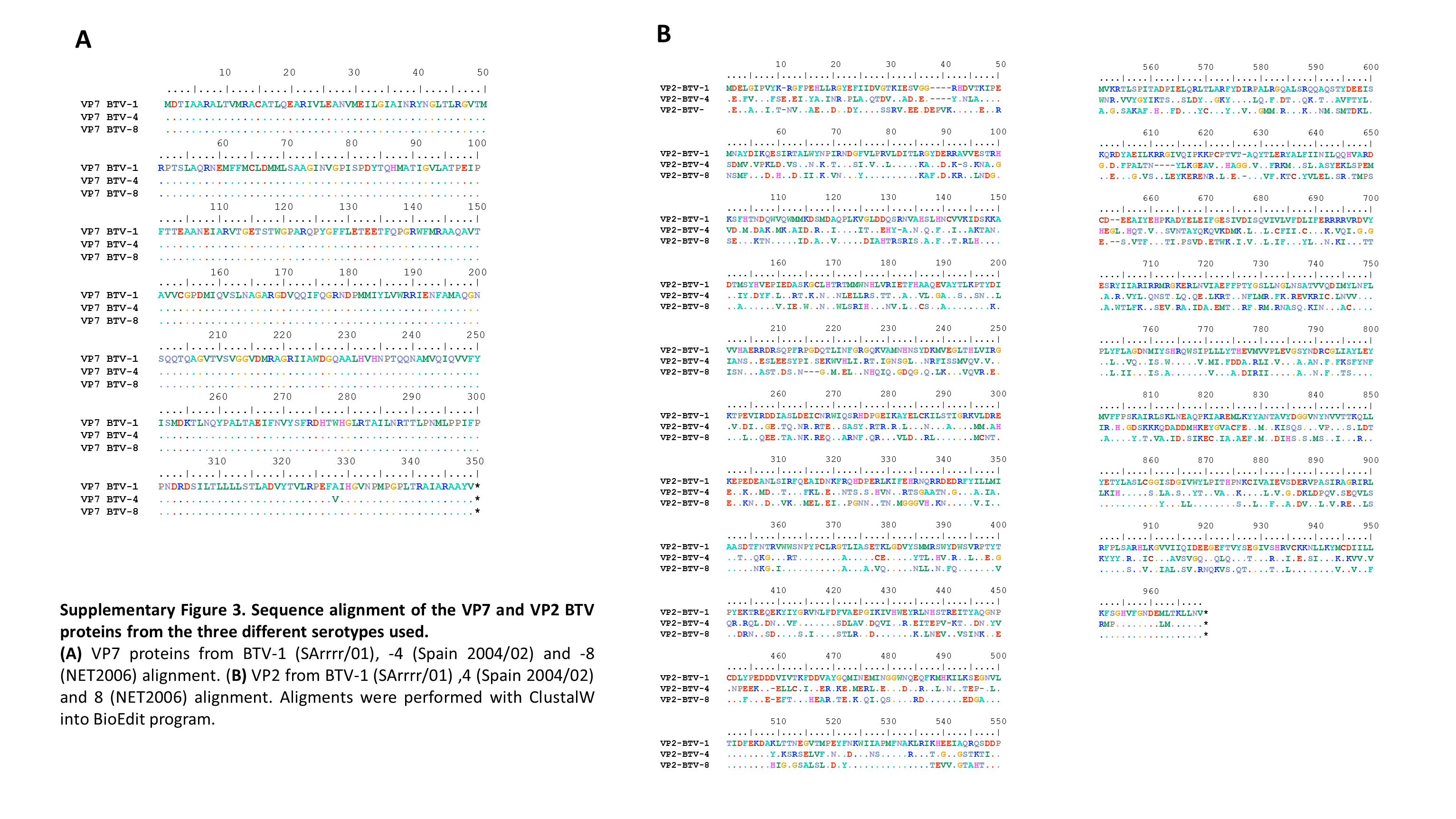

Supplement: Supplementary file 3 [file Image_3.TIF]
